# Proton Beam Therapy Combined with Intra-Arterial Infusion Chemotherapy for Stage IV Adenoid Cystic Carcinoma of the Base of the Tongue

**DOI:** 10.3390/cancers11101413

**Published:** 2019-09-22

**Authors:** Kanako Takayama, Takahiro Kato, Tatsuya Nakamura, Yusuke Azami, Takashi Ono, Motohisa Suzuki, Akinori Takada, Hisashi Yamaguchi, Ichiro Seto, Tatsuhiko Nakasato, Hitoshi Wada, Yasuhiro Kikuchi, Kenji Mitsudo, Nobukazu Fuwa, Masao Murakami

**Affiliations:** 1 Department of Radiation Oncology, Southern Tohoku Proton Therapy Center, 7-172, Yatsuyamada, Koriyama, Fukushima 963-8052, Japan; takahiro.kato@mt.strins.or.jp (T.K.); nakamurata@igtc.jp (T.N.); ya2ami@fmu.ac.jp (Y.A.); ono.takashi@qst.go.jp (T.O.); motohisa.suzuki@mt.strins.or.jp (M.S.); hisashi.yamaguchi@mt.strins.or.jp (H.Y.); ichiro.seto@mt.strins.or.jp (I.S.); tatsuhiko.nakasato@mt.strins.or.jp (T.N.); hitoshi.wada@mt.strins.or.jp (H.W.); y.kikuchi@mt.strins.or.jp (Y.K.); masao.murakami@mt.strins.or.jp (M.M.); 2 Department of Oral and Maxillofacial Surgery, Yokohama City University Graduate School of Medicine, 3-9, Fukuura, Kanazawa, Yokohama, Kanagawa 236-0004, Japan; mitsudo@yokohama-cu.ac.jp; 3 Department of Radiology, Mie University Hospital, 2-174, Edobashi Tsu, Mie 514-8507, Japan; a-takada@clin.medic.mie-u.ac.jp; 4 Department of Radiation Oncology, Ise red cross hospital, 1-471-2, Funae, Ise, Mie 516-8512, Japan; nobufuwa@ise.jrc.or.jp

**Keywords:** proton beam therapy, adenoid cystic carcinoma, intra-arterial infusion chemotherapy, the base of the tongue, chemoradiotherapy

## Abstract

Adenoid cystic carcinoma (ACC) is a very rare epithelial tumor of the salivary glands. Surgical resection is considered to be a standard therapy. However, the optimal treatment strategy for managing advanced cases has not yet been established. This study evaluated the efficacy and toxicity of proton beam therapy (PBT) combined with selective intra-arterial infusion chemotherapy (IAIC) using weekly cisplatin for locally advanced ACC of the base of the tongue. Between March 2009 and February 2018, 15 patients were treated. The median follow-up duration was 56 (range: 15–116) months. The 5-year local control and overall survival rates were 89% and 76%, respectively. With regard to late toxicities, grade 2 osteoradionecrosis was found in one patient and grade 5 pharyngeal necrosis was observed in one patient. Considering most cases were significantly advanced and inoperable, this therapy was effective in controlling the primary tumor, preserving function and maintaining the quality of life. Although improvements are needed to reduce adverse events, PBT in combination with IAIC can be a treatment option for locally advanced ACC of the base of the tongue.

## 1. Introduction

Adenoid cystic carcinoma (ACC) is a rare epithelial tumor of the salivary glands accounting for approximately 10% of all malignant salivary gland neoplasms. It represents approximately 1–2% of all malignant head and neck tumors [1]. The most common intraoral site for minor salivary gland tumors is the hard plate, followed by the base of the tongue [2], where up to 96% of all tumors are malignant and where ACC represents 30% of such cases [3]. ACC tumors are characterized by a rather slow but insidious growth pattern, a high propensity for perineural invasion and distant metastasis, and a pronounced ability to recur over a prolonged period. The diagnosis is delayed as it mainly progresses submucosally without symptoms. Therefore, 75% of ACCs of the base of the tongue show a progression of beyond stage T3 at the time of diagnosis [4].

The standard treatment for advanced ACC of the base of the tongue is complete surgical resection, such as total resection of the tongue and flap reconstruction. These kinds of surgical approaches require wide margins because of their extensive infiltration with perineural invasion and can result in the impairment of both swallowing and speech functions. ACC has been recognized as a radioresistant tumor. Radiotherapy has been considered for advanced T-stages or in the presence of positive microscopic margins as adjuvant therapy [5,6]. Some authors have suggested that the results of radiotherapy for advanced and inoperable ACC tumors indicate the worst outcomes. Furthermore, their local control rates were reported to be less than 50% [7,8,9]. There is no clear evidence on the subject of effective chemotherapy. Although definitive chemoradiotherapy has often been performed, the therapeutic effect is unfavorable [9].

Recently, a method of selective intra-arterial infusion chemotherapy (IAIC) via a superficial temporary artery (STA) was developed [10]. The concurrent chemoradiotherapy using X-ray therapy (XRT) and IAIC showed good treatment results as well as good functionality and aesthetics preservation in locally advanced oral squamous cell carcinomas [11]. Robbins et al. reported the efficacy of rapid intra-arterial supradose cisplatin once a week and concurrent radiotherapy (RADPLAT) for advanced head and neck cancer [12]. Cisplatin has a high anti-tumor effect and can be administered repeatedly while suppressing the adverse events by using a neutralizing agent. Even for the cisplatin-resistant tumor, the therapeutic effect is considered to be dependent on the concentration. Although it is well known that ACC is a chemo-resistant tumor, good treatment results with radical chemoradiotherapy with IAIC have also been reported for locally advanced ACC. Homma et al. reported that IAIC with concomitant radiotherapy is considered one of the treatment options for patients with inoperable ACC [13].

The particle beams provide a larger degree of relative biological effectiveness (RBE), and therefore, a higher probability of tumor control compared with that of XRT [14]. Particle beams also offer their own unique Bragg peaks and sharp lateral penumbras. As compared with conventional XRT, particle beams create an inherently three-dimensional conformal dose distribution, while minimizing doses delivered to the surrounding normal tissues [15]. Although good treatment results of particle beam therapy for the head and neck ACC including at the base of the tongue have been reported [16,17], the cases are still a few in number. 

The purpose of this study was to evaluate the efficacy and toxicity of proton beam therapy (PBT) combined with selective IAIC (PBT-IAIC) for locally advanced ACC of the base of the tongue.

## 2. Results

### 2.1. Patient Characteristics

Between March 2009 and February 2019, 15 patients with stage IV ACC of the base of the tongue were treated with PBT-IAIC. The patient characteristics are summarized in Table 1. A total of 15 patients fulfilled the inclusion criteria. The median age was 60 (range: 31–78) years, and there were five males (33%) and 10 females (67%) included. Of the 15 patients, six (40%) had cervical lymph node metastasis at the time of treatment initiation, five had lung metastases (33%), and six (40%) had unresectable disease. One of the seven patients with resectable disease was inoperable because of old age, whereas the remaining six patients refused to undergo surgery.

### 2.2. Compliance

All patients but one (93%) completed PBT and at least five cycles of IAIC (median: 7 cycles; range: 5–8 cycles). The treatment characteristics are summarized in Table 2. The median total dose of the biological effective dose (BED) of EQD_10/2_ was 70.3 (range: 62.6–73.8) Gy (RBE). The irradiation fields were prepared separately for the primary lesion and the cervical lymph nodes, respectively. The same dose was used in patients with overlapping irradiation fields. The median total cisplatin dose of all patients was 410 (range: 200–580) mg/body.

### 2.3. Response and Survival

**Figure 1:**
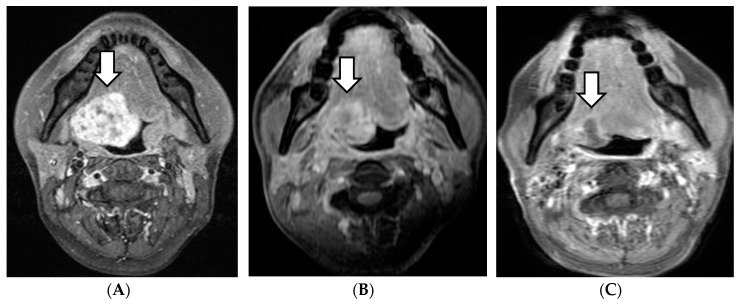
MRI of a patient, cT3N1MO case. The initial treatment effect was PR; thereafter, the tumor continued to shrink. (**A**) Before treatment. (**B**) After 3 months. (**C**) After 48 months. White arrow: initial or remaining tumor.

Four of the 15 patients had a complete response (CR), 10 patients experienced a partial response (PR), and one revealed stable disease within 6 months after therapy. The therapeutic effects of cervical lymph nodes metastases were CR in three cases, PR in two cases, and SD in one case. In the follow-up period, the primary lesions were reduced in all PR cases. In our study, the reduction of the primary lesions after treatment was relatively slow in the majority of patients, over a period from several months to several years (Figure 1).

The median follow-up duration was 56 (range: 15–116) months for all patients. Regarding the recurrence patterns, during the follow-up period, one patient developed a local recurrence at 36 months and one patient developed neck lymph node metastasis at 31 months (retreated with PBT). Both of these patients had lung metastases before treatment and refused salvage surgery. Additionally, four patients showed new lung metastasis. Three of five patients who had lung metastasis at diagnosis died from the progression of multiple lung metastases at 24, 51, and 76 months after treatment. One patient died from bleeding from pharyngeal necrosis at 28 months later, two patients died from other diseases at 68 and 98 months later, and nine patients remained alive at the conclusion of the follow-up (including three with lung metastases).

**Figure 2:**
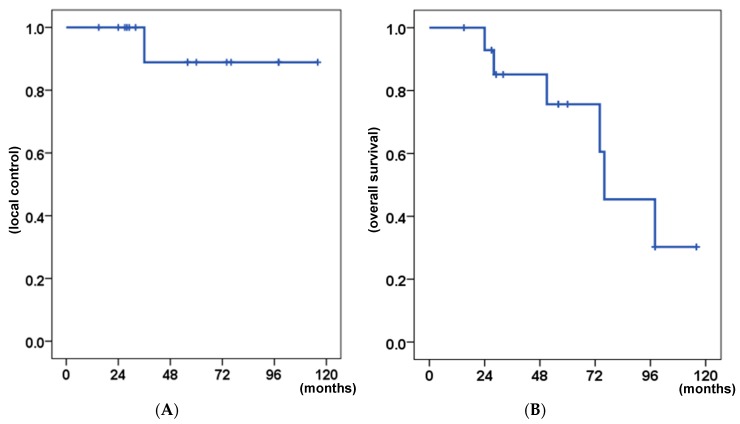
Kaplan–Meier curves of the clinical results for all patients. (**A**) Local control. (**B**) Overall survival.

The 5-year local control (LC) and overall survival (OS) rates were 89% and 76%, respectively (Figure 2). Table 3 shows the results of log-rank tests for the prognostic factors. None of the factors examined had a significant influence on LC in the univariate analysis. However, lung metastasis before treatment correlated with poor OS (*p* = 0.007).

### 2.4. Toxicities

**Figure 3:**
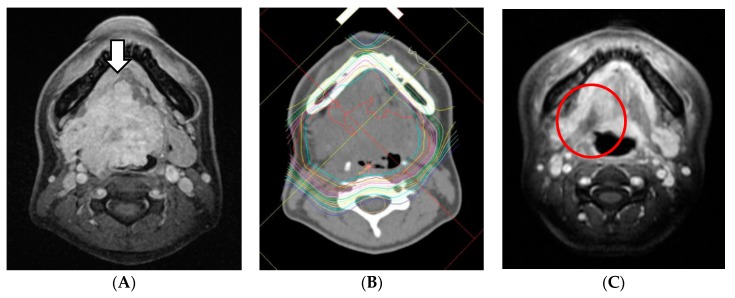
MRI of a patient, cT4aN0M1 (lung), before and after treatment. The tumor showed a CR but necrotic ulcer occurred. It was healed with conservative treatment. (**A**) Before treatment. White arrow: tumor. (**B**) The dose distribution of PBT. (**C**) After 2 months. Red circle: necrotic ulcer.

With regard to acute toxicities, those higher than grade 3 were as follows: oral mucositis in 12 patients (80%), dermatitis in three patients (20%), neutropenia in two patients (13%), and anemia in one patient (7%). Separately, grades 1 or 2 pharyngeal mucositis (ulcer) occurred in four patients within 2 months from the end of the treatment (Figure 3). With regard to late toxicities, grade 4 dysphagia and grade 5 pharyngeal necrosis were observed in one patient. No cases of irreversible dry mouth or mandibular osteomyelitis occurred (Table 4).

## 3. Discussion

To the author’s knowledge, this is the first report to evaluate the efficacy and toxicity of PBT-IAIC for the management of locally advanced ACC of the base of the tongue. In the present study; the 5-year LC and OS rates were 89% and 76%, respectively.

ACC of the base of the tongue is an extremely rare tumor, and the frequency has been estimated to be approximately 0.1% of all malignant tumors of the head and neck [9]. As ACC is a rare disease, there are no reports containing a significant number of cases on the clinical results of ACC of the base of the tongue. The treatments for ACCs most often combined wide resection with postoperative XRT. Previous studies have suggested possible treatments of ACC, including surgical resection, radiotherapy, chemotherapy, and combined therapy. Namazie et al. reported 14 patients with ACC of the base of the tongue treated with various methods. These patients’ reported 5-year OS rate was 79%, and the authors recommended combination therapy (surgery and radiation therapy (± chemotherapy)) for the treatment of advanced diseases [18]. Though ACC presents extensive infiltration with perineural invasion as compared with other malignant neoplasms, it is more difficult to fully remove, with frequently identified positive surgical margins [19]. Thus, surgical approaches for ACC require wide margins and can result in the impairment of speech and the swallowing function. Despite aggressive surgical resection, indolent local recurrences are common due to extensive local tissue infiltration and perineural spread. Metastasis to regional lymph nodes is uncommon, while distant metastasis to the lungs is more frequent. Huang et al. reported a potential distant metastasis risk in lesions primarily located in the base of the tongue [20]. Despite metastasis, however, long-term survival is common [21,22]. Although ACCs are generally regarded as relatively radioresistant tumors [23], locally advanced and inoperable cases treated by radiotherapy alone yield miserable results [5,6,7,8,9]. In more recent years, the improved treatment results have been reported with particle beam therapy for head and neck ACC. Koto et al. highlighted promising results among 18 patients with ACC of the base of the tongue treated with carbon-ion therapy (CIT), where the reported 5-year LC and OS rates were 92% and 72%, respectively [17]. Takagi et al. reported that 5-year LC rates of PBT and CIT for head and neck ACC were 75.5% and 77.7%, respectively, while the 5-year OS rates were 63.3% and 93.8%, respectively. They concluded that there were no significant differences between PBT and CIT in terms of OS, LC, and late complications. They reported that the LC rate of the proton beam alone was 75.5% and also reported that T4 classification was significantly associated with worse OS according to the multivariate analysis [16]. The present study treated more advanced cases and efforts to increase the therapeutic effect were needed. This study treated 15 patients with locally advanced ACC of the base of the tongue using PBT-IAIC and ultimately, the 5-year LC rate was 89%. Good therapeutic effects were observed following the use of PBT-IAIC for the local control of ACC. Although all patients presented stage T4, with the majority diagnosed with inoperable disease, PBT-IAIC for ACC of the base of the tongue was effective in controlling the primary tumor, preserving function, and maintaining the quality of life. The 5-year OS rate with this therapy was 76%, which is a good result considering that five of the 15 patients (33%) had distant metastasis prior to treatment. These findings suggest that PBT-IAIC might be equal to or better than conventional treatments.

Osteoradionecrosis (ORN) is a major problem in definitive radiotherapy for head and neck cancer. In previous studies, the incidence of mandibular osteomyelitis and ORN after postoperative radiotherapy in head and neck cancer has varied widely from 0.4% to 56%. Although the frequency was relatively high in past three-dimensional radiation therapy (3D-RT), in recent reports, the actuarial rate of ORN of the mandible in radiotherapy for oropharyngeal cancer was decreased to approximately 7% when using IMRT [24,25,26]. Particle beam therapy can better reduce the irradiated dose in the mandible as compared with XRT. Concerning the incidence of ORN with CIT for ACC of the base of the tongue, Koto et al. reported two patients in 18 (11%) developed grade 3 ORN of the mandible [17]. In this study, one patient of the 15 (7%) developed grade 2 mandible ORN but was healed by conservative treatment, and ORN as a late adverse event has not occurred. Tsai et al. concluded that there is a large difference in the incidence of ORN between 50-Gy and 60-Gy irradiation, and minimizing the percentage of mandibular volume exposed to 50 Gy may reduce the ORN risk [26]. Considering that patients are receiving a radiation equivalent to a median of 70 Gy, an incidence of ORN of 7% is considered acceptable. These results suggested that particle beam therapy may reduce the incidence of ORN even at high doses. In addition, osteomyelitis and ORN have been reported to be heavily involved in the oral environment [25]. Osteomyelitis and ORN are more likely to occur when teeth are in a poor state, caused by odontogenic infection. ACC is a relatively slow-growing tumor and can be improved upon by maintaining sufficient oral care before treatment and extracting teeth with poor prognoses.

Dysphagia is another significant side effect that may appear after radiation therapy in patients with oropharyngeal cancer. Previous studies have reported that 11% of patients demonstrated chronic dysphagia following radiation therapy for oropharyngeal cancer [27]. In this study, the rate of grade 3 or higher dysphagia as a late effect was 7%. Although the number of included cases was small, our results suggest that particle therapy may reduce dysphagia by minimizing the radiation field.

Pharyngeal ulcers sometimes occur after radiation therapy, and pharyngeal necrosis as a late disorder can be fatal. Previously, after treatment using CIT for ACC of the base of the tongue, in three of 18 cases (21%), a necrotic ulcer was reported but the ulceration and pain disappeared with tumor regression [17]. In this study, three cases of grade 2 ulcer formation and two cases of grade 3 ulcer formation occurred. The first treatment case developed grade 4 dysphagia and grade 5 pharyngeal necrosis due to delayed conservative treatment. This is because it was not possible to judge whether the ulcer that appeared in the base of the tongue was due to residual tumor or tissue necrosis. Since then, conservative treatment has been performed with great care, and thereafter all cases were cured.

In this study, five cases already had lung metastases at the time of the initial visit. These individuals were diagnosed as inoperable because of distant metastases. Although ACC is locally aggressive, it presents high recurrence levels and late metastasis due to slow clinical development, commonly leads to patient death between 10 and 20 years after the initial treatment [18,28]. If an increase in the primary lesions is largely associated with prognosis, the treatment may be meaningful even if patients have pulmonary metastasis. In the univariate analysis, lung metastasis before treatment was significantly associated with poor OS (*p* = 0.007). In this study, five out of 15 cases (33%) had lung metastases before treatment, and the OS rate may have been unusually decreased as a result. Despite the four cases of new additional lung metastases that occurred after treatment, our reported 5-year survival rate was 76%, which is a good result. Furthermore, in many malignant tumors, the accumulation of FDG is considered useful for determining the effects before and after treatment, and SCC examines it as an index for judging the treatment effect [29]. However, the SUV_max_ values and the difference in SUV_max_ values of FDG-PET/CT before and after treatment did not affect the prognostic factors in this study. It was suggested that the accumulation of FDG may not be useful for judging the therapeutic effect in the treatment of ACC.

The current study has some limitations that should be brought up. First, it was a retrospective and single-center study with a small patient sample, which therefore had inherent biases. However, the authors attempted to overcome this issue by including a homogenous study population of patients with only ACCs of the base of the tongue. However, it is a very rare tumor with very advanced patient cases. This is a study with a smaller sample size and a lack of previous studies on ACC. The statistical tests would not be able to identify significant relationships within the data set. A larger sample size could have generated more accurate results. This pilot study may be meaningful in future research. Second, the follow-up period was short. Therefore, the follow-up of the study cohort is still ongoing to further examine the long-term prognosis and late toxicities.

## 4. Materials and Methods

### 4.1. Ethics Statement

This retrospective study was approved by the ethics committee of Southern Tohoku Research Institute for Neuroscience (no. 339; approved on 21 September 2018). This study was conducted according to the principles of the Declaration of Helsinki.

### 4.2. Patients and Study Design

**Figure 4:**
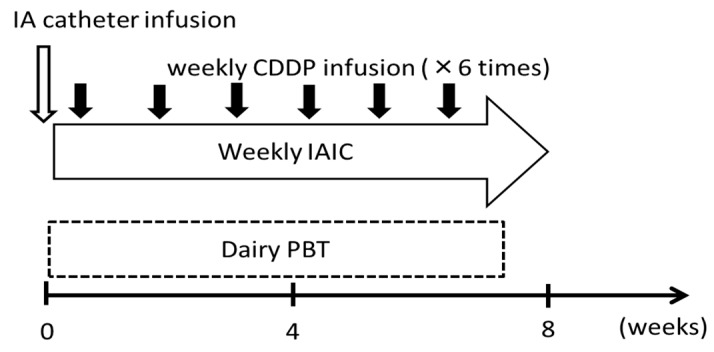
Treatment schedule. IA, intra-arterial; CDDP, cisplatin (20–40 mg/m^2^); IAIC, intra-arterial infusion chemotherapy; PBT, proton-beam therapy.

The eligible patients were as follows: histologically confirmed ACC of the base of the tongue; stage IV; no history of radiotherapy for a head and neck cancer; and follow-up duration of more than 12 months. The patients were required to have an Eastern operative Oncology Group performance status (EOCG-PS) of 0 or 1, a white blood cell count, >3500/μL; neutrophil count, >2000/μL; a platelet count, > 1 × 10^5^/μL; hemoglobin, >9 g/dL; and no severe dysfunction of the liver, kidney and heart. Tumor restaging was based on the Union for International Cancer Control TNM Classification of Malignant Tumors, eighth edition [30] by disease evaluation that included computed tomography (CT), magnetic resonance imaging (MRI), and fluoro-2-deoxy-D-glucose positron-emission tomography-CT (PET-CT). Written informed consent was obtained from all subjects prior to the initiation of treatment. The treatment schedules are summarized in Figure 4. All patients received daily PBT and weekly IAIC.

### 4.3. PBT

In the supine position, a thermoplastic head mask was immobilized to ensure high repositioning accuracy of the target, 1-mm CT slices and 3-mm MRI slices were obtained. For PBT planning, this study developed a CT-based three-dimensional treatment planning system (Xio-M; ELEKTA, Tokyo, Japan and Hitachi, Tokyo, Japan). The gross tumor volume (GTV), including the primary tumor and regional lymph node metastases, was defined by CT, MRI, and/or FDG-PET/CT. The clinical target volume (CTV) was defined as the GTV with a 3- to 5-mm basic margin in all directions, avoiding the mandible bone without tumor invasion. Then, the CTV expanded to included further areas suspected of tumor nerve infiltration. The planning target volume (PTV) was defined as the CTV plus a 3-mm margin to compensate for setup uncertainty.

The proton beams were arranged in the optimal angles to avoid excess-dose exposure to the normal tissue, adopting the dual-portal broad-beam method and multi-leaf collimators. Basically, the total PBT dose administered was 66 to 72.6 Gy (RBE) in 30 to 33 fractions, with 2.2 Gy (RBE) per fraction. Irradiation was performed five times a week using two or three portals of proton beams and energy levels of 150 or 210 MeV. The total dose at the isocenter was prescribed to cover 90% of the PTV. The proton-beam dose was defined as the physical dose multiplied by an RBE value of 1.1 and described in units of Gy (RBE). As there were multiple types of doses used in the treatment, the effects of PBT were comparable with those of BED. BED was calculated with the linear-quadratic model to compare the effects of the treatment and late complications with different fraction sizes and total doses. BED Gy (RBE) = total dose × (1 + dose per fraction/(α/β)). The α/β ratio was 10 Gy (RBE) for tumors and 3 Gy (RBE) for normal tissues. EQD_(α/β)/2_ signifies the equivalent dose as 2-Gy fractions for some value of α/β. EQD_(α/β)/2_ was calculated as follows: EQD_(α/β)/2_ = BED/(1 + 2/(α/β)) [31].

### 4.4. IAIC

Three-dimensional computed tomography angiography of the carotid artery was performed to determine the morphology of the tumor-feeding arteries. Catheterization from the STA was performed according to the method described by Fuwa et al. [10]. Briefly, an incision was made anterior to the ear on the affected side under local anesthesia to expose the STA, and a thin catheter was inserted from the STA into the lingual artery (LA) or external carotid artery (ECA). Cancers of the base of the tongue are typically surrounded by the region that is supplied by the LA. The facial artery, occipital artery, ascending pharyngeal artery, and innominate arteries branching from the main external carotid artery can sometimes be involved. Considering the blood flow, the catheter tip was selectively placed into the LA. However, to target the entire tumor spreading through the surrounding tissue, the tip of the catheter therefore was placed in a location where the anticancer agents were expected to flux through the entire tumor. When the primary tumor extends over the midline, the other catheter was inserted into the contralateral side to achieve bilateral arterial injection. The pigment injection test using indigotindisulfonate sodium, digital subtraction angiography, and flow-check MRI [32] can enable the catheter to be placed at the appropriate position and help detect tumors by confirming the enhancement of the feeding area form the catheter. The dose of cisplatin was 20–40 mg/m^2^ over 5 h, once a week, for four to six times. When the catheter was inserted selectively, the dose of cisplatin was set at up to 20 mg/m^2^. During the intra-arterial infusion of cisplatin, the cisplatin-neutralizing agent sodium thiosulfate was also administered intravenously at 8 g/m^2^ over 8 h, the dose was set in accordance with the dose of cisplatin.

### 4.5. Patient Assessments (Follow-up)

According to the Response Evaluation Criteria in Solid Tumors guidelines, Version 1.1., the clinical response was evaluated based on the results of the MRI performed 4 weeks after the treatment. All patients were observed at 3-months intervals for 1–3 years after therapy and at 6-months intervals thereafter. The acute reactions and late complications were evaluated according to the Common Terminology Criteria for Adverse Events, Version 4.0 [33].

The OS was calculated from the last day of PBT to the date of death or last confirmed date of survival. The LC was calculated from the last day of PBT to the date of local recurrence. The OS and LC curves were estimated using the Kaplan–Meier method, and the log-rank test was used for univariate analysis, including the age, sex, surgical indication, primary tumor size, irradiation volume, lymph node status, lung metastasis before treatment, PET SUV_max_, total radiation dose, and total dose of cisplatin on IAIC as independent variables. All *p* values were two-sided, and a *p* value of less than 0.05 was considered to indicate statistical significance. All statistical analyses were performed using IBM SPSS statistics version 22.0 (IBM Corp., Armonk, NY, USA).

## 5. Conclusions

PBT-IAIC was useful in establishing control of the primary tumor, preserving function, and maintaining the patient’s quality of life. Although it is necessary to consider the optimal dose in terms of the late effects relief, this treatment could be considered as a viable treatment option for managing stage IV ACC of the base of the tongue.

## Figures and Tables

**Table 1:** Patient characteristics.

Characteristic	*n* or Median (% or Range)
Number of patients	15
Age, years	60 (31–78)
Sex	
Male	5 (33)
Female	10 (67)
ECOG-PS	
0	10 (67)
1	5 (33)
T classification *	
T3	1 (7)
T4a	12 (80)
T4b	2 (13)
N classification *	
N0	9 (60)
N1	2 (13)
N2b	2 (13)
N2c	2 (13)
M classification *	
M0	10 (67)
M1	5 (33)
Stage	
IVA	9 (60)
IVB	1(7)
IVC	5 (33)
Location	
Right	9 (60)
Left	6 (40)
FDG accumulation before treatment	7.3 (1–12.5)
Reasons for not performing surgery	
Operabl	
Refusal	6 (40)
Old age	1 (7)
Inoperable	
Locally advanced	3 (20)
Distant metastasis	2 (13)
Locally advanced + distant metastasis	3 (20)

Abbreviations: EOCG-PS, Eastern Cooperative Oncology Group Performance status; FDG, fluoro-2-deoxy-D-glucose. * UICC, Union for International Cancer Control TNM classification, 8th edition.

**Table 2:** Treatment characteristics.

Characteristics	*n* or Median (% or Range)
GTV	50 (21–120) mL
PTV	160 (75–331) mL
Prescribed dose	
61.6 Gy (RBE)/28 fr.	1 (7)
66.0 Gy (RBE)/30 fr.	4 (26)
70.4 Gy (RBE)/32 fr.	4 (26)
72.6 Gy (RBE)/33 fr.	1 (7)
others	5 (33)
Total radiation dose	
BED_10_	84.4 (75.2–88.6) Gy (RBE)
BED_3_	119.1 (106.8–125.8) Gy (RBE)
EQD_10/2_	70.3 (62.6–73.8) Gy (RBE)
EQD_3/2_	71.5 (64.1–75.5) Gy (RBE)
Total dose of cisplatin on IAIC	410 (200–580) mg
Arterial injection vessels	
LA + ECA	6 (40)
LA	1 (7)
ECA	8 (53)
Nutrition	
PEG	6 (40)
Oral intake	5 (33)
Naso-gastric tube	4 (26)

Abbreviations: GTV, gross tumor volume; PTV, planning target volume; RBE, relative biological effectiveness; BED, biological effective dose; EQD_10/2_, equivalent dose as 2-Gy fractions for α/β = 10; EQD_3/2,_ equivalent dose as 2-Gy fractions for α/β = 3; IAIC, intra-arterial infusion chemotherapy; LA, lingual artery; ECA, external carotid artery; PEG, percutaneous endoscopic gastrostomy.

**Table 3:** Results of log-rank tests for prognostic factors.

Factors	No. of Patients	*p* Value	
		OS	LC
Age		0.790	0.264
<60 years	7		
≥60 years	8		
Sex		0.430	0.724
Male	5		
Female	10		
Surgical indication		0.720	0.264
Operable	7		
Inoperable	8		
Primary tumor size (GTV)		0.744	0.264
<50 mL	7		
≥50 mL	8		
Irradiation volume (PTV)		0.835	0.264
<160 mL	7		
≥160 mL	8		
Lymph node status		0.157	0.264
Negative	9		
Positive	6		
Lung metastasis before treatment		0.007 *	0.061
Negative	10		
Positive	5		
SUVmax value on PET		0.284	0.371
<7.3	8		
≥7.3	7		
Reduction of SUVmax after treatment		0.364	0.371
<5.9	7		
≥5.9	8		
Total dose; EQD_10/2_ (GyE_10/2_)		0.664	0.371
<70.3 Gy (RBE)	8		
≥70.3 Gy (RBE)	7		
Total dose of cisplatin on IAIC		0.607	0.264
<410 mg	7		
≥410 mg	8		

Abbreviations: OS, overall survival; LC, local control; GTV, gross tumor volume; PTV, planning target volume; SUV, standard uptake value; PET, positron-emission tomography; EQD_10/2_, equivalent dose as 2-Gy fractions for α/β = 10; EQD_3/2,_ equivalent dose as 2-Gy fractions for α/β = 3; RBE, relative biological effectiveness; IAIC, intra-arterial infusion chemotherapy. * *p* < 0.05.

**Table 4:** Adverse events (NCI-CTCAE v. 4.0).

Toxicity, maximum/latest	Grade, *n*				
	1	2	3	4	5
Mucositis oral	0/2	3/0	12/0	0	0
Dermatitis radiation	4/0	6/0	3/0	0	0
Neutropenia	0	6/0	2/0	0	0
Anemia	0	2/0	1/0	0	0
Platelet count decreased	0	1/0	0	0	0
Nausea	6/0	4/0	0	-	-
Acute kidney injury	0	0	0	0	0
Hepatobiliary disorders	1/0	0	0	0	0
Dry mouth	7/3	2/0	0	-	-
Dysgeusia	9/4	6/0	-	-	-
Laryngeal edema	3/0	0	0	0	0
Dysarthria	3/1	2/1	0	-	-
Dysphagia	4/2	2/1	2/0	1/1	0
Pharyngeal mucositis or ulcer	10/4	3/0	2/0	0	0
Pharyngeal necrosis	-	-	0	0	1/1
Osteonecrosis of jaw	0	1/0	0	0	0

Abbreviations: NCI-CTCAE v. 4.0, National Cancer Institute—Common Terminology Criteria for Adverse Events, version 4.0.; *n*, number.
